# Failure Analysis of a Humeral Shaft Locking Compression Plate—Surface Investigation and Simulation by Finite Element Method

**DOI:** 10.3390/ma12071128

**Published:** 2019-04-06

**Authors:** Iulian Vasile Antoniac, Dan Ioan Stoia, Brandusa Ghiban, Camelia Tecu, Florin Miculescu, Cosmina Vigaru, Vicentiu Saceleanu

**Affiliations:** 1 Department of Metallic Materials Science, Physical Metallurgy, University Politehnica of Bucharest, 313 SplaiulIndependentei, J Building, District 6, 060042 Bucharest, Romania; antoniac.iulian@gmail.com (I.V.A.); ghibanbrandusa@yahoo.com (B.G.); tecu_camelia@yahoo.com (C.T.); f_miculescu@yahoo.com (F.M.); 2 Department of Mechanics and Strength of Materials, Politehnica University of Timisoara, 1 Mihai Viteazul Avenue, 300222 Timisoara, Romania; cosmina.vigaru@upt.ro; 3 Faculty of Medicine, University Lucian Blaga of Sibiu, 2A Lucian Blaga Str., 550169 Sibiu, Romania; vicentiu.saceleanu@gmail.com

**Keywords:** LCP failure, FEM simulation, fracture morphology, fractography

## Abstract

A case study of a failed humeral shaft locking compression plate is presented, starting with a clinical case where failure occurred and an implant replacement was required. This study uses finite element method (FEM) in order to determine the failure modes for the clinical case. Four loading scenarios that simulate daily life activities were considered for determining the stress distribution in a humeral shaft locking compression plate (LCP). Referring to the simulation results, the failure analysis was performed on the explant. Using fracture surface investigation methods, stereomicroscopy and scanning electron microscopy (SEM), a mixed mode failure was determined. An initial fatigue failure occurred followed by a sudden failure of the plate implant as a consequence of patient’s fall. The fracture morphology was mostly masked by galling; the fractured components were in a sliding contact. Using information from simulations, the loading was inferred and correlated with fracture site and surface features.

## 1. Introduction

Humeral shaft fractures account for 1–3% of all fractures and 20% of the fractures of the humerus bone [1]. Among them, those which are located in the lower/upper third of the shaft represent more than 80% of the total cases, where 60% of the fractures occur in people over the age of 50. In over 60% of the cases the affected bone part was the middle third, with transverse and short spiral fractures [2,3]. 

The shaft of the humerus extends between the superior edge of pectoralis major muscle and the supracondylar distal humeral ridge. In the most frequent cases, the fracture of the humerus shaft is caused by a fall or a torsion traumatism at old age, or by a high energy traumatism at young age [4].

Due to its complex neurovascular anatomy formed by a complex network of muscles, nerves, and arteries, it is very important to perform a thorough examination of a fracture before undertaking surgical or non-surgical decisions. In the majority of cases, such injuries can be successfully treated non-operatively, due to the extensive muscle and soft tissue cover that can be used to splint the fracture. However, despite the success of non-operative treatments of humeral shaft fractures, there are several types of fractures which require an open reduction and internal fixation. The main decision stands in whether to consider a plate implant for the open reduction of the fracture or intramedullary nails [5,6].

The selection of the surgical approach is dependent on the fracture location and on the surgeon’s preference. Most frequently in recent years, middle-third shaft fractures have been approached antero-laterally, where osteosynthesis plates are used [7,8]. The outcomes for this are considered excellent, with a consolidation in more than 90% of the cases, a low rate of complications, and early re-entering in function.

The anterolateral or posterior placement of the fixation plate depends upon the fracture type and surgeon preference. At least six screws (preferably eight) have to be placed proximal and distal to the fracture site, especially in young patients [9].

The treatment of humeral shaft fractures can be associated with risk factors that lead to complications, including old age, living habits, any medical condition that impacts muscles, poor bone condition, or fracture-site-specific [10]—the type of trauma (low- or high-energy) that can affect the vascular system through its bone defragmentation. The vascular system can further impact the blood support to the fracture site and lead to a non-union situation in the end. 

Implant performance could be influenced by factors like the failure of the surgical procedure, such as plate fixation [11]; deviations from the physician prescription; high loads and high physical activity; interaction between the implant and the biological system; the material of the implant and screws, which can lead to inflammation followed by failure [12]; the design and length of the screws [13]; and the configuration of the screws with respect to the palate [14]. The number of free holes that the plate possesses at the end of the surgery can negatively influence the loading capacity of the system, due to the stress concentration effect that a free hole induces in the plate [15,16]. Also, the anatomical positioning of the implant and its geometry, together with the fixing procedure [17], can enhance system durability. Usually, failure of the implants occurs in the first three postoperative months, in 1–4% of the cases [18], due to complex mechanical and biological factors that generate corrosion [19]. Yet, one of the major complications of plate fixation is represented by a radial nerve injury. The radial nerve is vulnerable both during initial exposure and during the placement of the cortical screws. In addition, several authors [20] have reported neuropraxias attributed to excessive traction during fixation of the fracture. In all cases, these injuries were temporary and healed fully.

The failure of a 316 L proximal humerus internal locking system was investigated by Jason Ina et al. using energy dispersive X-ray analysis and electron backscatter diffraction [21]. The analysis reveals a set of clinical and mechanical reasons for implant failure. The mechanism of corrosion fatigue fracture, corroborated with mechanical overloading caused by using a humeral plate in ankle arthrodesis, were the major factors that generated failure. Furthermore, Farah Hamandi et al. [22] used FEM simulations and topographical SEM in order to perform a qualitative and quantitative failure analysis. By finite element analysis they validated the loading conditions and the stress distributions that led to failure.

The present study covers a FEM simulation conducted on an LCP in different loading scenarios, corresponding to regular daily activities, and an SEM analysis of the fracture site. The results are extrapolated towards a clinical case of implant failure and it aims to establish if the cause of the premature LCP failure, taking into account all the discussed potential factors, could also come from other directions and not only from patient not fully respecting the recovery program. An intact implant should be able to sustain high loadings that may occur in dynamic daily activities, if it is not weakened by high-cycle fatigue, which is less probable in such a short time after surgery. 

## 2. Materials and Methods 

### 2.1. Clinical Details and Circumstances of Implant Failure

**Figure 1:**
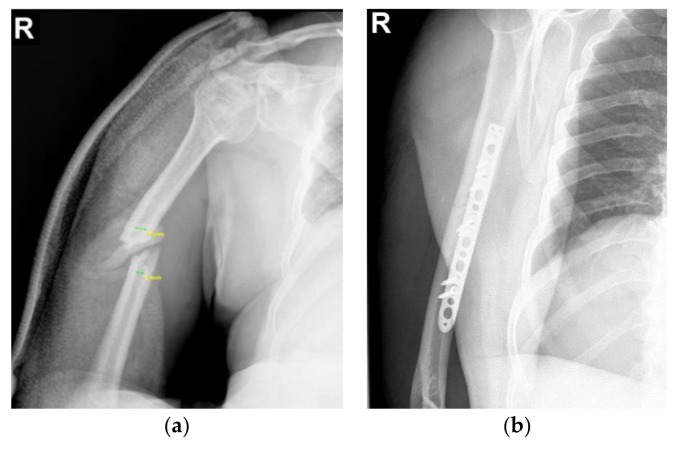
Clinical details: (**a**) X-ray comminuted fracture of midshaft humerus; (**b**) Post-operative X-ray with reduction and osteosynthesis with the locking compression plate (LCP).

**Figure 2:**
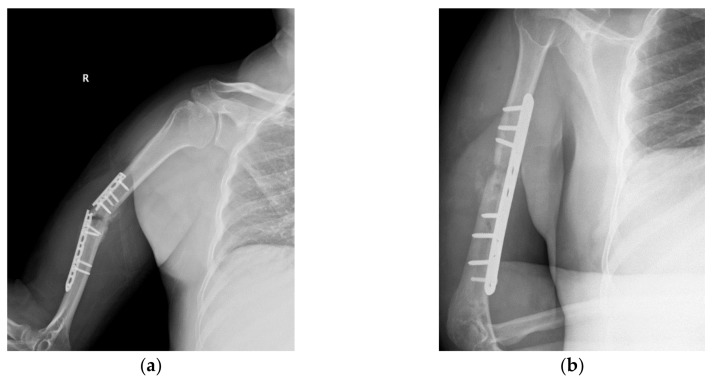
Clinical details: (**a**) X-ray: implant failure with subsequent plate breakage; (**b**) X-ray post-operative control after revision.

The patient is a 62-year-old male with a documented transverse fracture in the middle third humerus (Figure 1a), which was implanted using an LCP with eight screws. The screw configuration consists of five locking screws and three compression screws, according to Figure 1b. The patient was advised to follow a standard postoperative program with immobilization (sling) for the first three weeks, followed by three weeks of passive range of motion and a period of active range of motion after that. Nevertheless, two months after surgery he suffered a second injury, leading to an increased mobility of the arm, that produced a second fracture and consequently the plate failure. An X-ray examination revealed that he suffered a complete breakage of the implant, as depicted in Figure 2a.

A second surgery was performed. The initial LCP implant was replaced with a new implant, a dynamic compression plate (DCP), seven fixation screws, and an iliac bone graft addition. The surgical result can be observed in Figure 2b. The DCP is used as an internal fixator of the fractured tibia and/or femur bones (the biomechanics of the humeral shaft is similar to the majority of long bones). This is an accepted technique for the healing of broken bones. These implants in modern orthopedics have proven their efficiency in restoring the anatomical union of broken bones. However, there are some factors that may lead to implant failure, followed by repetitive surgeries: selection of the wrong implant type, excessive loadings during the osteointegration period, deviation from physician prescription, high physical activity or exercise, immature surgical procedures, infections, and biological interaction between the implant and the body.

### 2.2. Structural Analysis Using FEM

The finite element analysis was performed based on geometrical information provided by clinical data. Computed tomography (CT) images of the upper limb, possessing the broken LCP, were acquired and the DICOM files imported in MIMICS 10.1 (Materialise Inc., Leeuwen, Belgium) in order to generate the 3D reconstruction [23,24]. The raw volumes were digitally processed for further refined and mesh repairing using Geomagic Studio 9 (3D Systems, Morrisville, NC, USA). Following these steps, the geometry of the bone structure was reconstructed and prepared for numerical simulation. 

**Figure 3:**
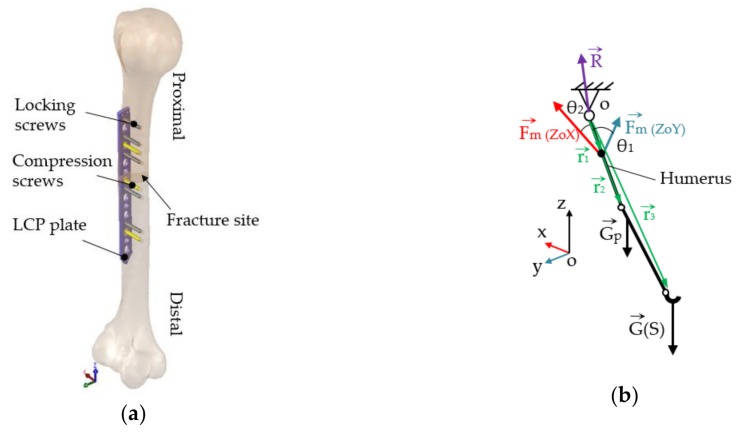
Geometric assembly and simplified static model: (**a**) Reconstructed humerus and LCP system fixed in place—internal lateral view; (**b**) Simplified body diagram of the upper limb used for computation of muscle forces.

The LCP and the two types of screws were designed in SolidWorks 2013 (Dassault Systèmes SE, Vélizy-Villacoublay, France) according to the geometries and dimensions measured on the CT images. By establishing the geometrical constraints between the bone elements, the LCP, and the screws, the geometric assembly was built (Figure 3a).

**Figure 4:**
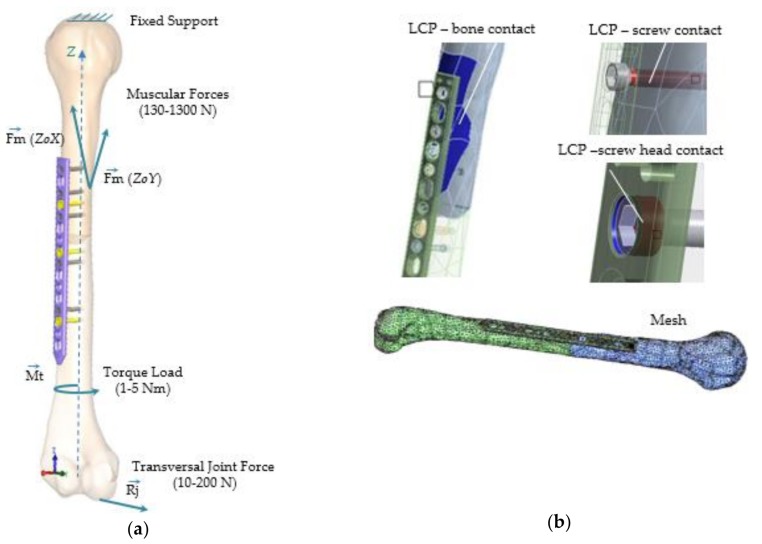
Structure loadings, constrains and contacts applied to the FEA model (all scenarios): (**a**) Loadings and boundary conditions; (**b**) Bonded contact definition: bone-plate, screw-plate, and screw-bone.

Any structural simulation requires the definition of boundary conditions. These refers to external loadings and weights that acts on a solid and also to geometrical elements that are considered to be fixed. The external reaction forces (joint force and muscular forces) produced by external loadings and weights were established using a simplified body diagram of the upper limb (Figure 3b). The unknown reactions were computed using static equilibrium equations written for the simplified model, Equations (1) and (2), taking into consideration four scenarios of external loading, uniform distribution of the segmental weight computed as a percentage of the total body weight, and length characteristics [25,26,27]. By solving the system of equations, muscular reaction forces were computed for 1, 3, 5, 8, and 10 kg external loads held in hand. According to the movement tendency, the external loads were counterbalanced by the corresponding muscle groups, as can be observed in Table 1 and Figure 4.

(1) $$\sum \vec{F}=\vec{R}+{\vec{F}}_{\text{m}}(ZoY)+{\vec{F}}_{\text{m}}(ZoX)+{\vec{G}}_{p}+\vec{G}(S)=0$$

(2) $${\sum \vec{M}}_{0}={\vec{r}}_{1}\times {\vec{F}}_{\text{m}}(ZoY)+{\vec{r}}_{2}\times {\vec{G}}_{p}+{\vec{r}}_{3}\times \vec{G}(S)+{\vec{r}}_{1}\times {\vec{F}}_{\text{m}}(ZoX)=0$$

The assembly comprises two bone elements, one LCP, five locking, and three compression screws. Studies by Gautier and Sommer [28] have shown that for a 12-hole plate, three screws in each fragment are advisable, keeping the plate screw density below 0.5–0.4. The assembled components are shown in Figure 3a, with the humerus in a flexed position.

In order to simulate the biomechanical behavior of the implanted bone assembly in various daily actions, four loading scenarios were used. Each scenario corresponds to a movement tendency and consequently to a muscle group activation (Table 1).

The 3D geometry was imported in finite element environment ANSYS 13 (ANSYS Inc., Canonsburg PA, USA), and material assignment, boundary conditions, contacts, and meshing were defined. The simulation was conducted several times by changing the loading conditions according to the predefined scenario, obtaining the regions of stress development.

The division of the continuum model was done using tetrahedral elements of constant size, comprising of 163,485 finite elements and 94,234 nodes, detailed in Table 2. All physical contacts between the surfaces of individual parts of the model were defined as bounded in order to simulate an osteointegration of two months’ time. For every element of the model material properties were added [29,30,31]. The study was performed using commercially pure (CP) titanium for the LCP and screws and equivalent bone properties [32,33,34,35] for the humerus, according to Table 3. Materials were defined as homogeneous, isotropic, and a linear elastic analysis was carried out [22]. All the results have to be considered on the assumption that all loads transferred from the bone to the plate directly and through the screws.

Based on the same simulation model and using the loading scenarios detailed in Table 1, a fatigue analysis was conducted in Ansys 13 (ANSYS Inc., Canonsburg PA, USA) [36]. The results of life (cycles to failure) are based on fatigue properties of the titanium (S-N curve) available [37,38,39] and on the following fatigue tool parameters: loading type was set to zero-based (R = 0); fatigue strength factor was equal to 1, meaning that there were no assumed imperfections or cracks at the surface of the palate; analysis type was based on the stress life method [22] and Goodman mean stress theory. The stress component selected for fatigue analysis was von Mises equivalent elastic stress [40].

### 2.3. Implant Retrieval and SEM Investigations

The failed implant was retrieved and studied to identify the failure mode and mechanisms. The investigations started with a generic macroscopic observation using Olympus SZX7 stereo-microscope. Higher magnifications on the fracture surface were achieved by a scanning electron microscope (SEM), ESEM metals XL 30 TMP. Energy dispersive spectrometry (EDS) was used to determine the chemical composition of the LCP.

## 3. Results and Discussions

### 3.1. FEM Simulation

**Figure 5:**
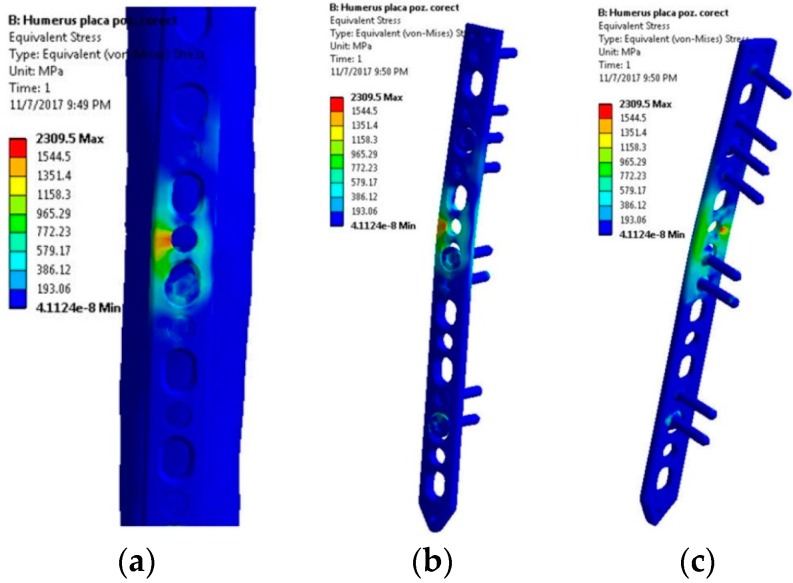
Stress distribution within the LCP when a 10 kg mass is lifted—flexion movement tendency: (**a**) Lateral view, bone-plate; (**b**) Lateral-front view, plate-screws; (**c**) Lateral-back view, plate-screws.

**Figure 6:**
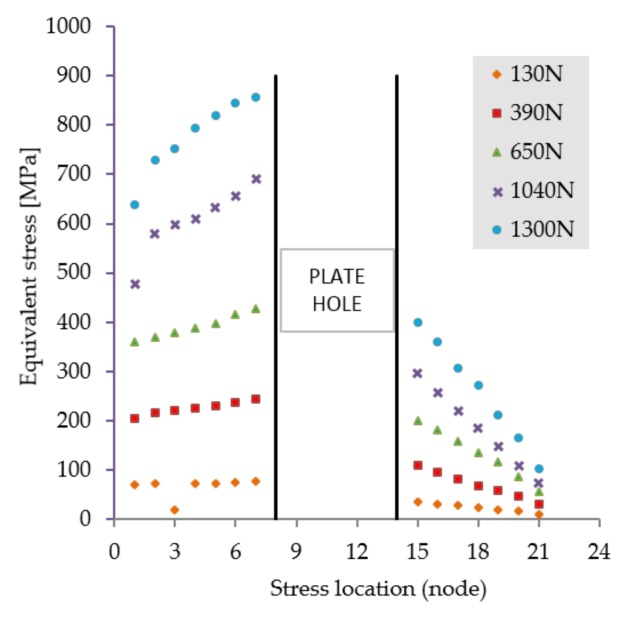
Stress distribution in the critical region. Scenario I.

The first scenario implies a flexion tendency of the humerus by activation of the anterior deltoid muscle, while at palm level the following weights are acting discretely: 1, 3, 5, 8, and 10 kg. The stress distribution presented in Figure 5 reveals high stresses at the hole positioned in the immediate vicinity of the bone fracture. Regardless of the weight values input, the insertion and direction of loading, together with the complexity of the assembly [41,42], cause a non-uniform and asymmetric stress distribution to appear in the plate. The plate is subjected to bending. Depending on loading values, von Misses stresses vary in magnitude according to the plot shown in Figure 6. 

In the second scenario, the loading is exerted by humerus abduction, while the same weight values are acting at palm level. The plate is in the posterior position with respect to the humeral tuberosity, thus the stresses are below the values of scenario I.

**Figure 7:**
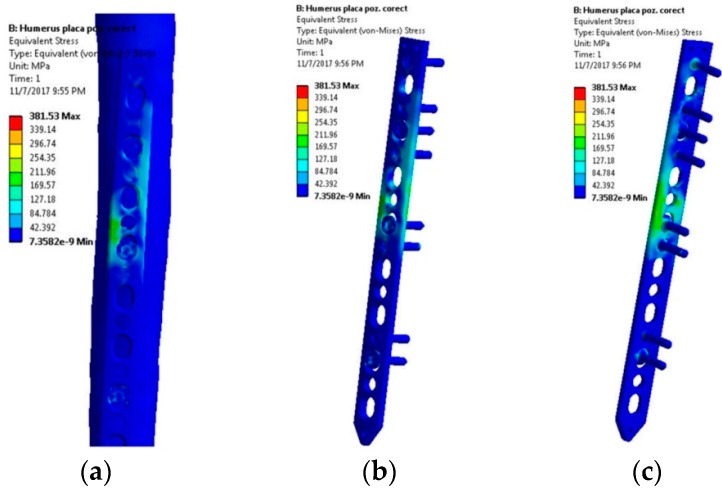
Stress distribution within the LCP when a 10 kg mass is lifted—abduction movement tendency: (**a**) Lateral view, bone-plate; (**b**) Lateral-front view, plate-screws; (**c**) Lateral-back view, plate-screws.

**Figure 8:**
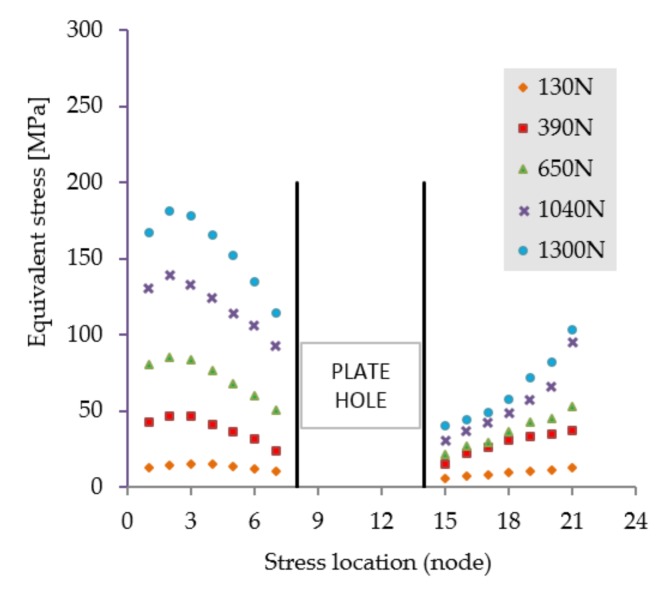
Stress distribution in the critical region. Scenario II.

The stress distribution in the LCP shown in Figure 7 suggests a dominant tensile stress manifesting an even distribution. Regardless of the external weight, in this loading scenario the plate will not fail, as the equivalent stress values indicate in Figure 8.

**Figure 9:**
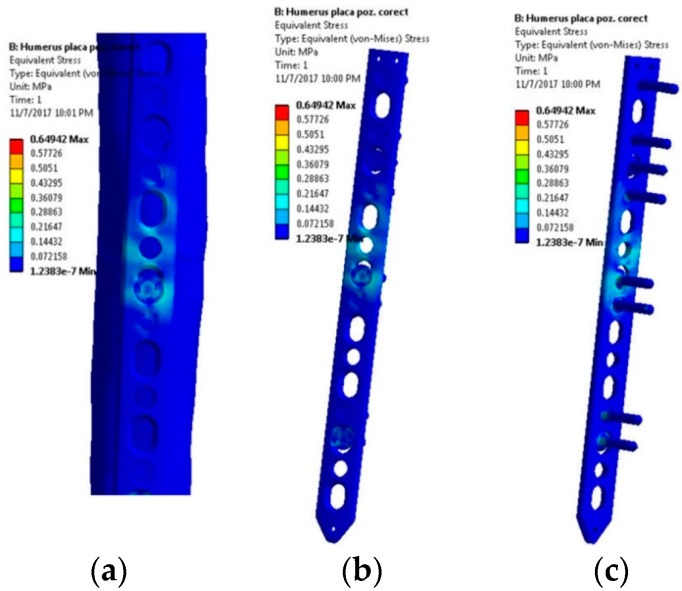
Stress distribution within the LCP when a 5 Nm torque is applied—rotation movement tendency: (**a**) Lateral view, bone-plate; (**b**) Lateral-front view, plate-screws; (**c**) Lateral-back view, plate-screws.

**Figure 10:**
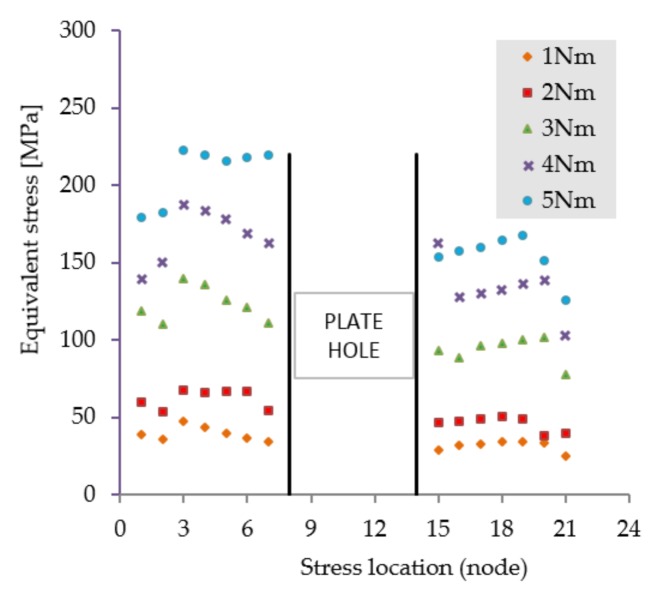
Stress distribution in the critical region. Scenario III.

Another frequent movement of the upper limb is the rotation of the arm, accomplished by the rotator cuff and deltoid muscles. The load was input as a torque vector around the Z axis, with a constant value and application point on the distal epiphysis. The torsion effect induces uniform stresses distribution in plate, that are presented in Figure 9. When loading 5 Nm of torque on the humerus, plate failure is unlikely, as the equivalent stress show in Figure 10.

In scenario IV a push/pull action on the radius is simulated. Considering a 90° flexion angle between the radius and humerus, a reaction force appears in the elbow joint. Due to the angulation of the bones, the reaction force acts perpendicular to the longitudinal axis of the humerus, generating a bending effect on the plate. The scenario was performed for reaction forces of 10, 30, 50, 100, and 200 N.

**Figure 11:**
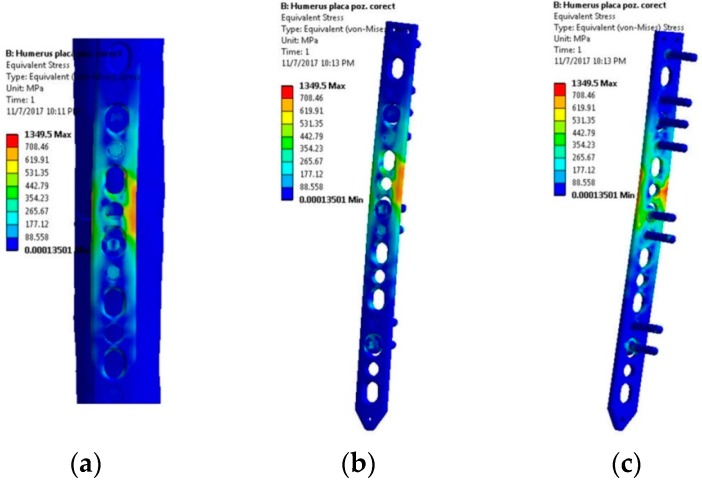
Stress distribution within the LCP when a 200 N transversal joint force acts: (**a**) Lateral view, bone-plate; (**b**) Lateral-front view, plate-screws; (**c**) Lateral-back view, plate-screws.

**Figure 12:**
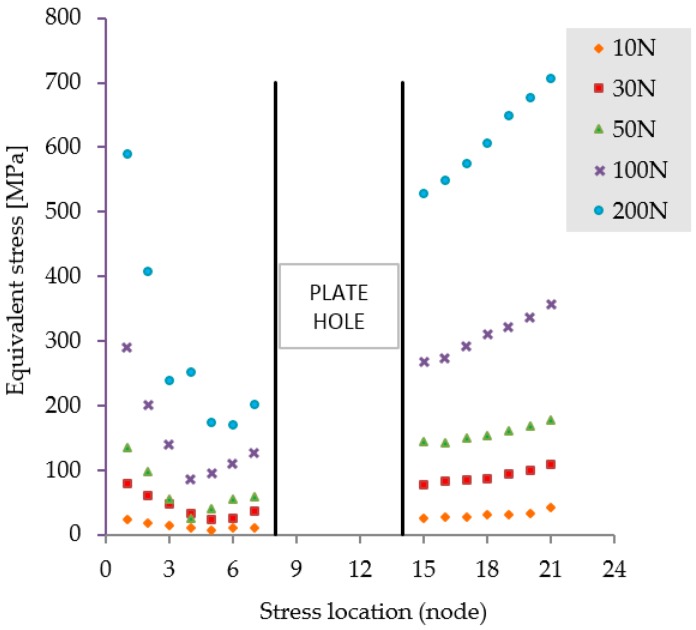
Stress distribution in the critical region. Scenario IV.

The posterior positioning of the plate with respect to the humerus and the direction of the reaction force lead to high stresses on the top surface of the plate and lower values on the opposite face (Figure 11). In Figure 12, stresses in the critical region are presented in relation to the loading values. 

According to the simulations, a load larger than 100–120 N will generate stresses that exceed the yield strength of CP titanium (377 MPa depending on processing) [29], which will subsequently lead to implant failure.

It must be stated that the study can be applied solely to LCPs made of CP titanium when no load bearing of the bone fragments is considered. Due to this aspect, and taking into account that no soft tissue was considered, we can affirm that the real stress values in LCPs will be lower, as the soft tissue and bone fragments will contribute to the stress flowing from the hand to the body trunk and finally to ground. 

Following the loading scenarios, it can be stated that the LCP would fail if the humerus was flexed while holding weights above 50 N. The failure mode would be bending—push/pull actions which generate reaction forces above 100 N in the elbow joint would cause implant failure, similar to the first scenario. A bending failure aspect would be expected in the retrieved implant—when the humerus is purely abducted there is a very small chance of failure, since the fracture site tends to close. The stresses here are mainly becoming tensile stresses. In torsion, no sudden failure would occur given the torque values used for simulation. Regardless of loading type, the same region is under heavier stress every time—the hole near to the bone fracture site.

**Figure 13:**
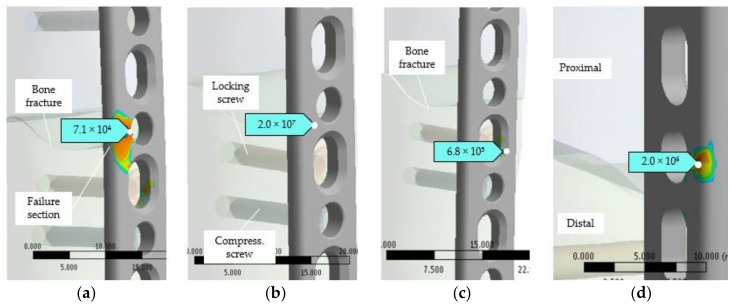
Fatigue life in critical section of the plate: (**a**) Scenario I, 1040 N loading; (**b**) Scenario II, 1300 N loading; (**c**) Scenario III, 5 Nm loading; (**d**) Scenario IV, 100 N loading.

Based on the same simulation model and loading scenarios, a fatigue analysis was conducted. The FEA fatigue simulation results are presented in Figure 13, where a representative picture of the LCP life for each loading scenario can be observed. The plate life for all loading values in each scenario is presented in Table 4.

**Figure 14:**
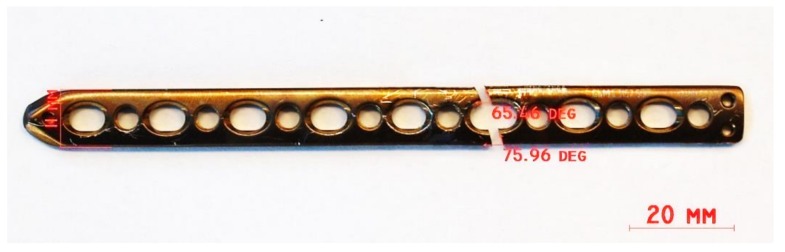
The retrieved LCP implant.

**Figure 15:**
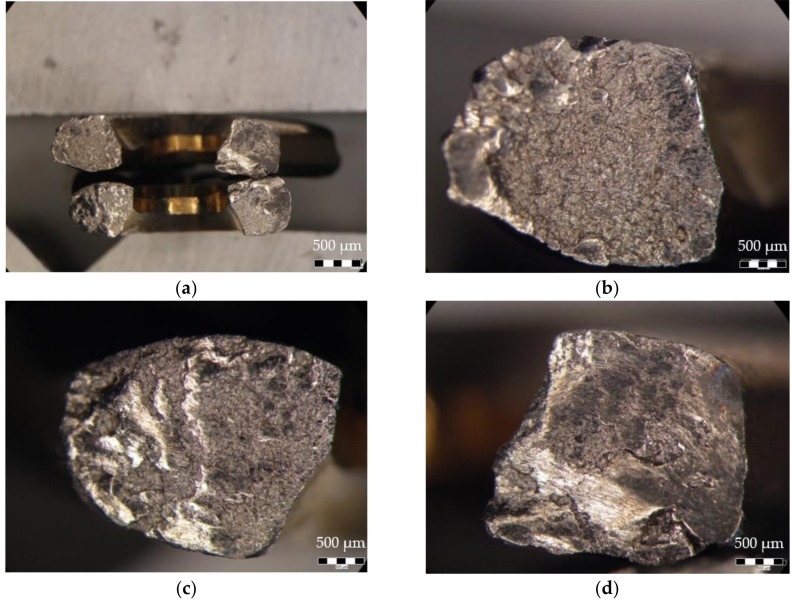

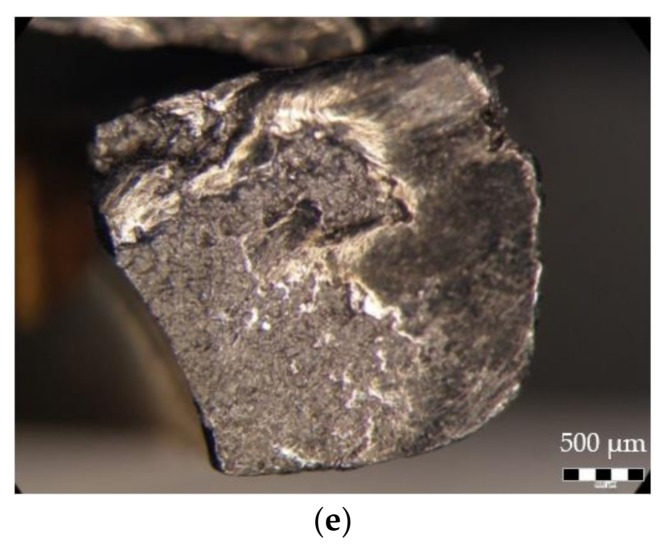
Fracture surface observed by stereomicroscopy: (**a**) overall fractographic image; (**b**) proximal side-posterior wall; (**c**) distal side-posterior wall; (**d**) proximal side-anterior wall; (**e**) distal side-anterior wall.

The minimum cycles to failure occurred naturally in the section of high stresses, which are currently in the vicinity of the plate hole that is located near to the bone fracture site. The equivalent elastic stress obtained by static simulation and the critical areas indicated by fatigue analysis are validated by the visual and fractographic examinations (Figure 14 and Figure 15). The failure occurs in the same spot as the simulation predicts.

The maximum allowable stress for pure titanium in order to stay on the endurance (fatigue) limit of 10^7^ cycles is equal to or less than 320 MPa [38,39], no matter the type of loading. The presence of stresses above this limit will finally cause plastic deformation and premature failure. 

As the simulation predicts, excepting scenario II where the fatigue limit was not exceeded for any loading values that we used, the rest of the scenarios may cause the fatigue failure of the palate, if a certain number of cycles and loading values are exerted (Table 4).

All the results were within the limitation of the assumption, which stated that all directly applied loads and reaction forces transfer from bones directly to the screws and plate, without taking into consideration the soft tissues that exist in a real environment and the micromechanical interface problems.

### 3.2. Implant Retrieval and Analysis

The retrieved implant is shown in Figure 14. This was measured using a digital caliper, resulting in the following overall dimensions: 11 mm width × 2.8 mm thickness × 133 mm length. 

The first observations show heavy scratch marks around the fracture zone, which occurred during implant retrieval and were produced by the surgical devices used for surgical retrieval. No corrosion features were observed by investigation with the naked eye.

Another interesting feature was the apparent straight-line failure at the 6th hole counted from the proximal end of humerus. The fracture line forms a 76° angle on the right and a 65° angle on the left side in relation to the longitudinal axis of the LCP. Three possibilities of failure may occur. The first failure mode consists of a bending failure of the right side of the plate, followed by an overloading failure on the left side. This implies that the first failure occurs on a section that has the highest moment of inertia (strong axis of the plate). The second failure mode may occur by bending along the weak axis of the plate. In this case the two sides will break at the same time. The third failure mode can be brittle torsional failure, produced by a torque applied on the longitudinal axis of the plate. This case also produces a one-step failure. By looking at the results of the numerical simulation and connecting those with the observational one we can affirm that sudden failure of the plate may be possible in bending on the strong axis of the plate. This kind of loading can be achieved in scenarios I and IV while the loafing exceeds 100 N. A sudden torsion failure is not likely to happened for daily routine loads up to 5 N·m. Fatigue fracture, on the other hand, may occur in all loading scenarios through crack propagation modes (opening, in-plane shear, or out-of-plane shear) [43], as we will describe in the following section.

#### 3.2.1. Fracture Surface Investigation by Optical Microscopy

For the fractographic investigation an Olympus SZX70 stereomicroscope was used. Using a metallographic cutter, samples were obtained from the fracture surfaces of the retrieved plate. 

On the fracture surfaces shown in Figure 15a, obvious signs of galling with friction welded material can be seen, mostly near to the margins. The surfaces were in sliding contact under compressive forces for a prolonged time, with the true fracture features being obscured by fretting.

The fracture surface in Figure 15b shows galling in the proximal side-posterior wall region followed by a wide area with a crystalline aspect revealing a sudden failure. It was inferred that the crack grew in the posterior-anterior direction of the plate.

In Figure 15c, the opposite surface to the one shown in Figure 15b, galling also appears on the exterior region of the plate, suggesting a crack path from distal side-posterior wall towards distal side-anterior wall. The presence of several large voids is a sign of a ductile fracture, caused probably by sudden loading.

The fracture surface shown in Figure 15d reveals a significant amount of galling. The morphology, although most features obscured, still resembles the one for a fatigue failure. The crack origin location can be inferred in the top right corner where there is a feature similar to a “blue spot” [44], a fracture aspect which was observed in stress corrosion cracking in a NaCl environment. Given this aspect, an NE–SV crack growth direction was considered. Sudden failure occurred when the section was severely reduced, and the surface showed a crystalline aspect.

On the fracture surface shown in Figure 15e, galling can be observed and a significant proportion of the fracture area reveals a crystalline aspect, suggesting a brittle fracture.

The fracture morphology on the four investigated surfaces describes the following mode of failure: the plate failed first by fatigue, the right-side revealed traces of fatigue striations and a small crystalline area. The galling covered a large portion of the surface, showing that after first failure the left side of the plate bore the majority of the load.

On the left side a fatigue mechanism could also be inferred, but the galling obscures most of the specific features. The fracture surface shows mixed modes of failure—overload ductile failure, where voids and dimples are present on the two opposing faces consequence of the second injury and the crystalline aspect specific to a brittle failure.

Based upon the morphology of the fracture the loading scenarios of the plate can be inferred. The right side failed by torsion fatigue, while the left side failed by bending fatigue with the final fracture caused by sudden overloading. 

The post-surgery loading scenario of a patient is a very personal issue. Statically high loadings are caused by heavy work, cycle loadings are caused by repetitive motions [45], and highly dynamic loadings are caused by falling or other impacts, all of which may occur.

#### 3.2.2. Fracture Surface Investigation Using SEM

**Figure 16:**
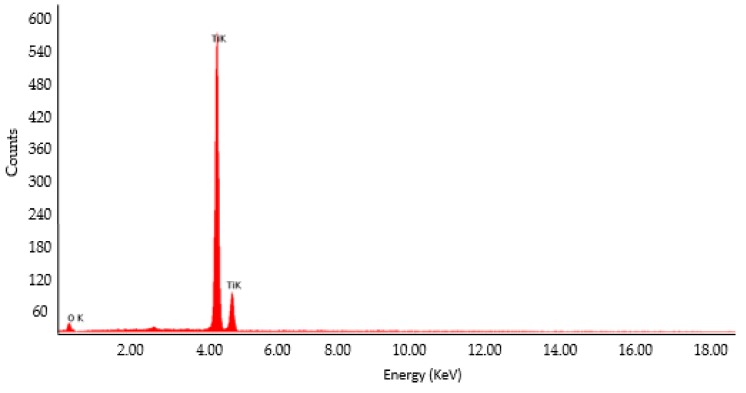
EDS spectra showing Ti and O specific peaks.

The fracture surface was observed using an SEM, and a chemical composition of the bone plate was studied using Energy-dispersive X-ray spectroscopy (EDS). The spectra shown in Figure 16 confirmed the presence of commercially pure titanium and oxygen, dissolved in the interstices in the LCP.

On the fracture surface an attempt was made to locate the fracture origin and identify crack growth and fracture mode. The identification of the crack origins and their development and union offer valid information regarding the failure mode of the structure [46,47]. The results shown in the following paragraphs were cumbersome to interpret because of fracture surface fretting.

**Figure 17:**
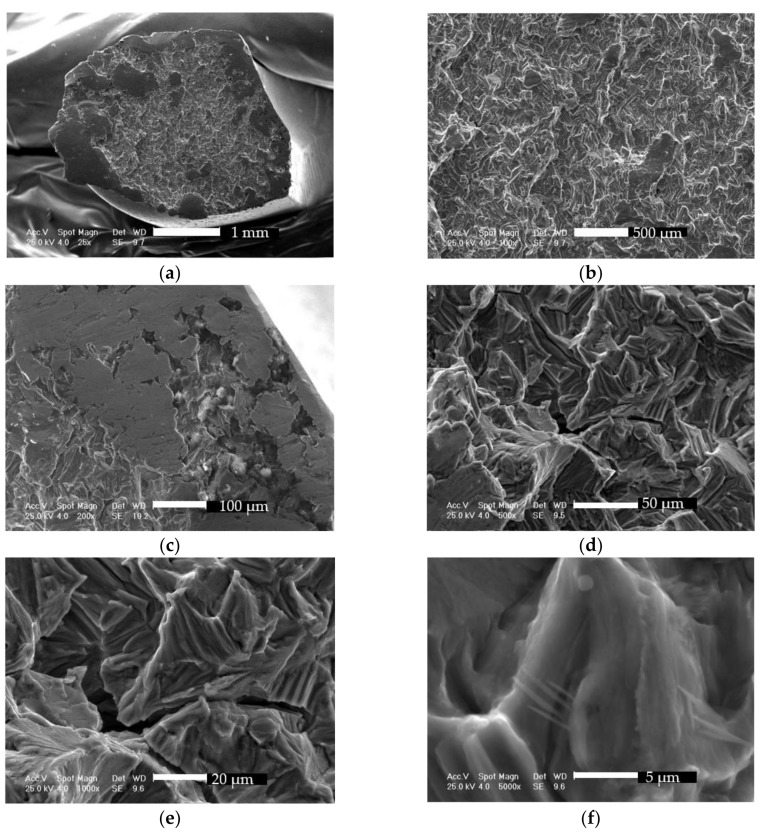
Scanning electron micrographs showing fracture morphology: (**a**) overall aspect of the fracture surface at 25×; (**b**) intercrystalline and transcrystalline fracture features at 100×; (**c**) sliding marks on galling features at 200×; (**d**) intercrystalline and transcrystalline fracture features at 500×; (**e**) fine cleavage steps on cleavage facets at 1000×; (**f**) fine cleavage steps on cleavage facets at 5000×.

The fracture surface shown in Figure 17a was thoroughly investigated in various sites at different magnifications in order to clearly determine failure and fracture mode. First observations were the lips, which appeared as a consequence of torsion failure. The galling features were magnified in Figure 17c where sliding marks can be seen, with an orientation which confirms a load in torsion. The crack origin was thought to be located at the exterior corner of the plate at the sixth hole. Surface reduction and torsion loading caused a sliding contact, which in turn caused galling. 

The center region, inspected at different magnifications (Figure 17b,d), reveals intercrystalline and transcrystalline fracture features, specific to fatigue failures in titanium [48,49], along secondary cracks. The feather markings, an array of fine cleavage steps on cleavage facets pointing back in the direction of the local crack, can be clearly seen in Figure 17e,f.

**Figure 18:**
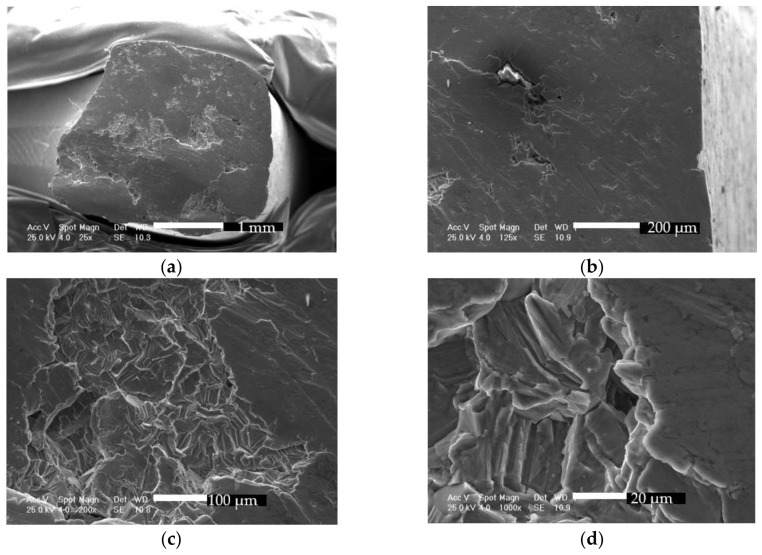
Scanning electron micrographs showing fracture surface morphology on first failure region: (**a**) first failed section at 25×; (**b**) sliding marks and lips at 125×; (**c**) typical brittle failure of the central region at 200×; (**d**) typical brittle failure of the central region at 1000×.

Studying the part which failed first, shown in Figure 18a, the galling covers most of the surface. Again, lips which suggest torsion failure can be observed, while in Figure 18b, at higher magnifications, the sliding marks and lips show the relative movement of the facets. The central region reveals typical brittle failure aspects with cleavage, details of which are presented in Figure 18c,d. Fracture origin could have been identified by backtracking the rough faceted fracture features/cleavage steps, coarse microstructure [50,51], and the fatigue striations with cracks which point to initiation site, but most features were obscured by galling.

## 4. Conclusions

Given the implant geometry and implantation site, limiting values for weight handling were established and the stress concentrations determined. In the numerical simulation, the region which was susceptible to failure was the wall section of the plate hole near to the bone fracture, regardless of loading scenario. 

The equivalent elastic stress obtained by static simulation and the critical areas indicated by fatigue analysis are validated by the visual and fractography examinations

The explanted LCP failed in two stages. The first failure occurred by fatigue in torsion (scenario III), while the final fracture also occurred by fatigue, most likely in bending (scenario IV). The final fracture was caused by overloading of the small remaining section. 

The stressed regions predicted by numerical simulation have essential value in inferring failure mode, since most of the fracture surface morphology was affected by fretting.

The patient did not follow doctor prescriptions. The activity conducted in the recovery period was intense (in loading value and frequency), causing premature failure of the plate. 

Given these results, a new method for LCP placement and/or fixation by avoiding the presence of the hole in the close vicinity of the bone fracture site remains to be studied, in order to achieve better implant performance.

## Figures and Tables

**Table 1:** Loading scenarios and boundary conditions (graphically depicted in Figure 4a).

Loading Scenario	Movement Tendency	Muscle Activation	Loadings F_m_, M_t_	Force/Moment Insertion	Fixed Support
I	Humerus flexion	Anterior deltoid	130; 390; 650; 1040; 1300 (N)	Deltoid tuberosity of humerus (YoZ)	Humeral head
II	Humerus abduction	Middle deltoid	130; 390; 650; 1040; 1300 (N)	Deltoid tuberosity of humerus (XoZ)	Humeral head
III	Humerus rotation	Rotator cuff + anterior and posterior deltoid	1; 2; 3; 4; 5 (N·m)	Long. axis of humerus (Z)	Humeral head
IV	Extension/flexion	Biceps/triceps –joint reaction	10; 30; 50; 100; 200 (N)	Distal head of humerus (YoZ)	Humeral head

**Table 2:** Mesh statistics for individual parts.

Assembly Parts	Elements	Nodes
LCP	19,122	35,662
Proximal humerus	31,875	52,012
Distal humerus	29,916	50,408
Locking screw	1181	2284
Compression screw	1941	3693

**Table 3:** Mechanical properties of the materials used in the FEM simulation.

Properties	Humerus Bone [31,32]	Titanium CP [29,30]
Young Modulus in tension	15.3 GPa (equivalent)	105 GPa
Poisson’s Ratio	0.31	0.37
Density	1.85 g/cm^3^	4.51 g/cm^3^
Compressive Yield Strength	-	450 MPa
Tensile Strength, Yield	-	377–520 MPa
Tensile Strength, Ultimate	-	440 MPa
Elongation to Break	-	18%
Reduction of Area	-	35%

**Table 4:** Life of the LCP according to each scenario.

**Loading Scenario I (Humerus Flexion)**					
Force (N)	130	390	650	1040	1300
Cycles to failure	>2.0 × 10^7^	2.0 × 10^7^	6.8 × 10^6^	7.1 × 10^4^	0
**Loading Scenario II (Humerus Abduction)**					
Force (N)	130	390	650	1040	1300
Cycles to failure	>2.0 × 10^7^	>2.0 × 10^7^	>2.0 × 10^7^	>2.0 × 10^7^	2.0 × 10^7^
**Loading Scenario III (Humerus Rotation)**					
Torque (N·m)	1.0	2.0	3.0	4.0	5.0
Cycles to failure	>2.0 × 10^7^	>2.0 × 10^7^	>2.0 × 10^7^	1.3 × 10^7^	6.8 × 10^5^
**Loading Scenario IV (Extension-Flexion)**					
Force (N)	10	30	50	100	200
Cycles to failure	>2.0 × 10^7^	>2.0 × 10^7^	2.0 × 10^7^	2.0 × 10^6^	0
